# Detecting Instability in Animal Social Networks: Genetic Fragmentation Is Associated with Social Instability in Rhesus Macaques

**DOI:** 10.1371/journal.pone.0016365

**Published:** 2011-01-26

**Authors:** Brianne A. Beisner, Megan E. Jackson, Ashley N. Cameron, Brenda McCowan

**Affiliations:** 1 California National Primate Research Center, University of California Davis, Davis, California, United States of America; 2 Department of Anthropology, The Pennsylvania State University, University Park, Pennsylvania, United States of America; 3 Department of Population Health & Reproduction, School of Veterinary Medicine, University of California Davis, Davis, California, United States of America; University of Alabama, United States

## Abstract

The persistence of biological systems requires evolved mechanisms which promote stability. Cohesive primate social groups are one example of stable biological systems, which persist in spite of regular conflict. We suggest that genetic relatedness and its associated kinship structure are a potential source of stability in primate social groups as kinship structure is an important organizing principle in many animal societies. We investigated the effect of average genetic relatedness per matrilineal family on the stability of matrilineal grooming and agonistic interactions in 48 matrilines from seven captive groups of rhesus macaques. Matrilines with low average genetic relatedness show increased family-level instability such as: more sub-grouping in their matrilineal groom network, more frequent fighting with kin, and higher rates of wounding. Family-level instability in multiple matrilines within a group is further associated with group-level instability such as increased wounding. Stability appears to arise from the presence of clear matrilineal structure in the rhesus macaque group hierarchy, which is derived from cohesion among kin in their affiliative and agonistic interactions with each other. We conclude that genetic relatedness and kinship structure are an important source of group stability in animal societies, particularly when dominance and/or affilative interactions are typically governed by kinship.

## Introduction

Stability in biological systems has been described as the persistence of regularities, and evolved mechanisms are necessary to promote or maintain this stability [1]. In primate societies, stability may be exemplified by the life span of the social group which typically extends beyond the life span of any individual group member. Competitive interactions among group members are inevitable, because conspecifics seek out similar resources (i.e., mates, food, alliance partners). The persistence of stable social groups in primate societies indicates that, despite the inevitable costs, group members gain a net benefit by living in a group. Social groups must, therefore, have ways of mitigating these costs, and thereby maintaining stability. Here we investigate the factors that influence the persistence of social groups, using rhesus macaques as a model species, by identifying those factors or circumstances which result in the opposite: instability.

### Detection of Group Instability

Stable social groups are those that persist through time in spite of competition that regularly arises among group members. Among wild primate groups, a reduction in group stability may lead to group fission [2], and in captive groups of primates, group instability may result in increased aggression among group members and possibly the dissolution of the group's hierarchy [3], [4] because group fission is often not possible in captivity. Group fission and severe aggression are likely symptoms of instability which result from the absence of underlying structures or mechanisms that typically maintain group stability. A complete understanding of stability in animal social groups requires not only detection of the symptoms of instability but discovery of the underlying source of that instability.

### Mechanisms of Group Stability

The mechanisms that contribute to group stability may depend on a number of different factors, such as conflict resolution or reconciliation [5], conflict interference by third parties [6], or group size and composition [7]. Precisely which factors play a role in group stability may be dependent upon the social system of a given species. For example, Flack and colleagues [6], [8] recently investigated conflict management behavior as a potential robustness mechanism, which serves to prevent the outbreak of very severe or uncontrolled aggression, within a captive group of pigtailed macaques (*Macaca nemestrina*). Conflict management by third parties, also called policing, involves impartial intervention upon others' conflicts resulting in termination of the conflict. Flack and colleagues found that temporary removal of conflict managers resulted in an increase in the rate of biting and intensity of aggression, thereby destabilizing the group [6]. The act of policing, however, appears to be performed by a small subset of powerful individuals [9] which is dependent upon a highly skewed power structure within the group (social power is a measure of group consensus that the individual has the ability to successfully challenge other individuals), suggesting that conflict management may only be a robustness mechanism for societies with a similarly skewed power structure.

### Kinship as a Mechanism of Group Stability

The mechanisms which promote group stability have yet to be identified for most animal societies, including the study species, rhesus macaques. Although conflict management appears to be a stabilizing mechanism in pigtailed macaque society, the highly skewed social power structure required to give some individuals sufficiently high social power with which to police the rest of the group may not be present in most societies. We suggest an alternative source of stability: kinship.

Kinship has long been thought to contribute to group and matriline cohesion in primate groups, although this assumption has rarely been demonstrated empirically. According to kin selection theory, most altruism and cooperation occurs between close genetic relatives because the cost to the actor is offset by the fitness benefit gained through genes shared with the recipient by common descent, thereby maximizing the actor's inclusive fitness [10]. In fact, the opportunity to cooperate with kin may be a primary selective force in the evolution of group-living among primates [11]. In many primate species, both dominance [12], [13], [14], [15], [16] and affiliative relationships are patterned by degree of kinship [17], indicating that kinship lends an organizational structure to the group. Therefore, we suggest that a degradation of kinship ties within matrilines, via a decrease in average genetic relatedness, may reduce matrilineal and group stability by degrading the organizational structure of social relationships among group members. Furthermore, because kinship structure is present in many different taxa [18], [19], [20], [21] genetic relatedness is a potential source of group stability for animal societies in general, including humans [22], [23], [24].

### Social network theory as a method for detecting group instability

Stability in a biological system is a higher-level outcome which arises from the interactions among lower-level components within the system. Social network theory (SNT) is therefore an ideal method for investigating the emergence of stability from interactions among group members. In recent years, biologists have increasingly used SNT to detect higher-level properties of biological systems from dyadic interactions among components [25], [26], [27], [28], [29]. Furthermore, SNT has also been successfully applied to the investigation of social group stability. Flack and colleagues used social network analyses in their investigation of conflict management as a robustness mechanism in pigtailed macaque social niche construction [8]. Absence of the policing mechanism was associated with reorganization of social niches, characterized by individuals forming smaller and less diverse networks, and showing a lower degree of integration within the group network. Additionally, McCowan and colleagues [3] have shown that increased fragmentation in displacement networks was associated with higher levels of aggression and greater likelihood of severe aggressive social overthrow.

### Instability at the matrilineal level

#### Grooming relationships

Grooming has long been known to serve a social function in primate societies, thus social grooming is one potential way for individuals to cope with competition and preserve group stability. First, grooming reduces tension and stress by lowering heart rate among those being groomed and lowering cortisol levels among both groomers and those being groomed [30], [31]. Secondly, grooming may be used to establish or maintain important relationships, either as an exchangeable commodity in biological markets [32], [33] or as reconciliation to repair damaged relationships [34]. Finally, grooming may be a primary means of promoting group cohesion [35], [36]. For these reasons, we investigated the stability of matrilineal grooming networks and the effect of average matrilineal stability on overall group stability.

Social groups of rhesus macaques consist of clusters of maternally related females called matrilines, and females show a preference for associating with kin. Grooming and aid in fights are patterned by degree of kinship, creating a matrilineal structure to agonistic and affiliative relationships [23], [37]. Thus, a breakdown of this matrilineal structure may weaken relationships among kin, which may in turn cause group-level instability. According to the maternal transmission hypothesis, kin-bias develops and persists via social transmission through the mother [38]. Sisters recognize each other as close kin by associating with a common mother who connects them. Over time, as these mothers succumb to predation, old age, or illness, the matriline becomes more genetically fragmented, and the mothers that once provided a social connection between sisters are gone. As a result, affiliative ties may weaken among matriline members, producing fragmentation in matrilineal affiliation networks. The absence of such mothers may further result in a greater average group-level matrilineal fragmentation as the group gets older, particularly in captive groups where fissions are not possible. Additionally, the presence or absence of the most recent maternal common ancestor of all matriline members (i.e., the matriarch) may play a significant role in the degree to which more distant kin affiliate with one another.

#### Agonistic and dominance relationships

Rhesus macaques are classified as the most despotic of the macaques [39], meaning they are characterized by severe aggression, highly asymmetrical dominance interactions, and a greater emphasis on kinship compared to other species of macaques. In particular, the influence of maternal kinship on group structure results in inheritance of maternal rank, which creates a hierarchy in which entire matrilines outrank other matrilines [14], [40]. Females aid kin in fights more than non-kin, and close kin are helped more frequently than distant kin [23], [37], producing a kin bias in female alliances that is important in the maintenance of matrilineal rank [12], [41]. Given this matrilineal organization, it is likely that instabilities in the group will first appear as instabilities of matriline ranks.

Matrilineal ranks might become destabilized if there is instability regarding the relative ranks among matrilines or among members of the *same* matriline. Both types of instability could originate from a low average relatedness coefficient among members of a matriline. First, a low average relatedness coefficient within a matriline indicates that the matriline is composed of a greater proportion of distant kin dyads than close kin dyads. Since agonistic alliances among kin are patterned upon degree of relatedness [23], this low average degree of relatedness may lead to fewer alliances among kin, which may in turn destabilize matrilineal dominance relations. Second, distant maternal kin may fight among themselves in effort to increase their individual rank. Indeed, among Japanese macaques (*Macaca fuscata*), lower-ranking matriline members given the opportunity to outrank a more dominant female (in the absence of the dominant female's kin) will sometimes ally with the dominant female to outrank her own kin [42].

### Aim and practical approach of the study

The goal of this study was to investigate the factors that influence the persistence of social groups, using rhesus macaques as a model species, by identifying those factors or circumstances which result in the opposite: instability. We calculated measures of instability at the matriline-level in seven groups of captive rhesus macaques. We investigated whether increased fragmentation within a matrilineal pedigree (lower average coefficient of relatedness among matriline members; absence of the matriarch) is associated with a greater number of communities per matrilineal grooming network, which may be an indication of instability within the matriline. We further investigated whether fragmentation within a matrilineal pedigree is associated with instability of matrilineal dominance using four measures of aggressive behavior at the matriline level: (1) the proportion of aggressive dyadic interactions initiated by a matriline using intense aggression, (2) the proportion of fighting events in which a matriline participates where the initiator and recipient are kin (3) the proportion of fighting events in which the matriline participates and in which intense aggression is involved where the initiator directs intense aggression at members of her own matriline, and (4) the frequency of wounding/injury received per matriline. Finally, in order to assess whether matrilineal-level instability influences group-level instability, we investigated whether a group-level average of matrilineal fragmentation is associated with the average wounding rate per group and the age of the group. In total, we used these analyses to evaluate the potential role of kinship structure, via genetic relationships, as a mechanism of stability in rhesus macaque social groups.

## Methods

### Ethics statement

All research reported in this manuscript adhered to the recommendations in the Guide for the Care and Use of Laboratory Animals of the National Institutes of Health, the laws of the United States government, and the recommendations of the Weatherall report, “The use of non-human primates in research”. All research subjects were housed in large social groups in half-acre outdoor enclosures to provide for their psychological well-being. The methodological approach was purely observational and involved no experimental or invasive treatment of the animals. All occurrences of illness or injury among study subjects were immediately reported to and treated by CNPRC veterinary staff, and all efforts were made to ameliorate suffering. This project was approved by the University of California, Davis Institutional Animal Care and Use Committee, protocol #11843.

### Study Site and Groups

The study was conducted at the California National Primate Research Center (CNPRC) in Davis, CA from June 2008 through November 2009. The subjects of this study were 48 matrilines from seven groups (Groups 1, 5, 8, 10, 14, 16 and 18) of rhesus macaques housed in 0.2 ha enclosures (Table 1). Minimum matriline size for inclusion in this study was five adults (3 years and older).

**Table 1:** Characteristics of study groups.

Group	Matrilines^a^	Mean (range) matrilineal coefficient of relatedness	Group size mean (range)	Observation period
1	11	0.29 (0.18−0.39)	176.5 (167−182)	Jun. – Nov. 2009
5	6	0.16 (0.11−0.27)	137.1 (127−148)	Jun. – Nov. 2008
8	8	0.20 (0.13−0.35)	160.1 (147−169)	Jun. – Nov. 2008
10	5	0.13 (0.09−0.18)	164.4 (150−175)	Jun. – Nov. 2009
14	6	0.17 (0.14−0.22)	108.3 (105−110)	Jun. – Nov. 2008
16	6	0.11 (0.08−0.13)	150.3 (141−158)	Jun. – Nov. 2008
18	6	0.18 (0.12−0.23)	197.9 (170−210)	Jun. – Nov. 2009

a Only matrilines having five or more members were analyzed.

All enclosures were similar in having ten A-frame houses, multiple suspended barrels, swings and several perches. Groups were fed a standard monkey chow diet twice per day at approximately 0700 hours and again between 1430 and 1530 hours. Fresh fruit or vegetables were provided twice per week.

Rhesus macaques in this outdoor colony were managed with a minimal level of disturbance, and individuals of each group were free to interact with one another as they chose. Disturbances within the enclosure were typically limited to daily morning health checks, two round-ups per year to conduct health examinations on all animals and removal of injured or sick animals for medical treatment.

### Sampling methods

Two observers (primary observers: BAB and MEJ) recorded both affiliative and aggressive interactions among members of 48 matrilines in seven groups to evaluate the degree of fragmentation in matrilineal grooming networks, measured as the number of communities per groom network [43], [44]. Each group was observed from 0900 h–1200 h and 1300 h–1600 h, four days per week, on a 4-week rotating schedule. Groups 5, 8, 14, and 16 were observed June through November 2008, and Groups 1, 10, and 18 were observed June through November 2009, which yielded 6 weeks of observation (144 hours) per group.

An event sampling design was used to collect data on agonistic interactions for approximately 6 hours per day. Agonistic interactions were recorded as an ordered series of dyadic interactions. Both aggressive and submissive behaviors were categorized in increasing levels of severity. Aggression included threat, vocal threat or threat and follow, lunge or mild slap, chase <3 meters, chase >3 meters or grapple, bite <5 seconds, chase and bite <5 seconds, and bite >5 seconds. Submission included silent bared teeth display (SBT), turn away, turn away with SBT, move out of arms' reach, move out of arms' reach with SBT, run away <3 meters, run away <3 meters with SBT, run away >3 meters, run away >3 meters with SBT, prolonged scream, crouch (animal stops resisting aggression and gives up, i.e. during mobbing events), and crouch with SBT. Intense aggressive interactions included bite <5 seconds, chase and bite <5 seconds, and bite >5 seconds.

Scan samples of dyadic interactions of grooming and contact-sitting were conducted every half hour. Affiliation scan samples were discontinued when aggressive events occurred. A total of 5767 grooming interactions were recorded (427–1290 per group) and 12,250 fighting events (1521–2175 per group) involving 29,849 aggressive dyadic interactions. Each fighting event consisted of one or more sequential aggressive dyadic interactions, involving two or more individuals. Affiliative and aggressive interactions were recorded for both males and females 3 years and older.

The heart of the matter of matrilineal cohesion is the definition of a matriline. The boundary of matriline membership is not necessarily defined by the presence of a matriarch, as two halves of a matriline whose matriarch is gone may still regard one another as kin, particularly if sisters have a strong relationship or the matriarch has not been gone long. However, the precise number of connecting females (such as the matriarch) that must be absent before separate branches of a matriline no longer regard one another as kin is not known. Among our study groups, defining matriline boundaries solely by descent from a matriarch that is present results in little variation in the average coefficients of relatedness, and actual kinship relationships appear to be discounted. Therefore, individuals were considered part of the same matriline if they could be traced back to the same female genetic common ancestor at the time of group formation. The oldest groups (10 and 16) were formed in 1976 (matrilines span 5–7 generations) and the youngest group (1) was formed in 1995 (matrilines span 3–5 generations). Males in these captive groups cannot disperse, and were therefore available to interact with their maternal kin and contribute to matrilineal cohesion. As natal males in these study groups do regularly interact (groom, form alliances) with their maternal kin [45], matrilines included maternally related males as well as females. Additionally, both males and females groom opposite sex partners during sexual consortships, and inclusion of these relationships in the network would not reflect matrilineal cohesion. Therefore, we excluded all instances of consortship grooming. Consortships were defined as a male-female pair that maintained almost constant contact throughout the day (grooming, huddling, mounting) during the breeding season, and both the male and female showed interest in maintaining contact with each other.

All study subjects were born in captivity and all subsequent births recorded, thus all maternal kin relationships were known. Paternal kin relationships, whereby paternal siblings or half-siblings form special relationships, were not taken into account. Although paternal kinship has been found to influence social behavior in rhesus macaque societies [46], maternal kinship is certainly the stronger organizing principle in rhesus macaques.

Relative matrilineal ranks were determined from behavioral management staff records of weekly observations of displacements and aggressive interactions and were supplemented by observations from this study. All members of the same matriline generally held the same rank. However, in cases where some matriline members held a different rank from the rest of their family, the matrilineal rank assigned was the rank held by the majority of the matriline members.

### Social Networks

Social networks of grooming interactions were visualized for each matriline (matriline size range: 5–27; mean  = 11.04) using UCINET 6.247[47] and the igraph package for the R statistical computing environment [48], [49]. Nodes represent individuals and ties represent grooming relationships. The grooming sociomatrix included all grooming interactions among matriline members plus grooming between matriline members and non-matriline members because indirect connections among matriline members which exist as a result of common direct connection to a non-matriline member may contribute to overall matrilineal cohesiveness. Network fragmentation was measured using the walktrap.community algorithm, which detects dense subgraphs within a network (called communities) by using random walks [44]. Community structure may be detected for multiple different partitions of the network into communities, and a modularity score,Q, is calculated for each partition [43], [44]. The partition at which Q is maximized is regarded as the most satisfactory division of the network into communities, where each community has strong within-community connections and weak between-community connections. If there were two local maxima for Q, the split having the smaller number of communities was chosen.

To determine whether random processes could have created the observed community structure, we constructed random networks for each matriline and compared their community structure with the observed networks. Each random network was created by simulating a set of edges for all possible dyads; the probability of edge presence was equal to the proportion of all possible edges observed in the real networks. One thousand simulations were run for each matriline. Inspection of the simulated random networks revealed that few of the observed matrilineal networks had a community structure that could have been produced by random processes. The proportion of simulated networks having the same community structure as the observed network was less than 5% for 31 matrilines, 5–9% for 11 matrilines, and greater than 10% (range: 13–82%, mean  = 31%) for six matrilines. We conclude from these comparisons that the observed network structure is not better explained by random processes.

### Statistical Analyses

We analysed the data using linear and generalized linear mixed-effects regression models [50]. Models were fit to the data for seven dependent variables. At the matriline level (N = 48 matrilines): (1) counts of communities per matrilineal groom network, (2) the proportion of aggressive dyadic interactions initiated by a matriline using intense aggression, (3) the proportion of fighting events in which a matriline participates where the initiator and recipient are kin, (4) the proportion of fighting events in which the matriline participates and in which intense aggression is involved and the initiator and recipient are kin, and (5) the frequency of wounding/injury received per matriline. At the group level (N = 7 groups): (6) the mean matrilineal average relatedness per group and (7) the mean rate of wounding per group. We ran a series of models for each dependent variable and used Akaike's Information Criterion (AIC) scores to select the best fit model, i.e., the model with the lowest AIC score. Following the recommendation of Burnham and Anderson [51], AIC scores were corrected for small sample size (N/K <40 for some models) and nested models having a difference in AIC score less than or equal to two (ΔAIC ≤2) were considered equivalent. A random effect for group was included in all models.

A generalized linear mixed-effects regression model (Poisson distribution) was fit to the counts of communities per matrilineal groom network using a robust estimator (aka Huber variance) of the covariance matrix [52]. Since Huber variance robustness to non-independence, over-dispersion, or under-dispersion may be achieved at the cost of decreased robustness for the finiteness of the number of clusters [53], we re-ran our analyses using a program which allows the t-distribution to be used in Huber variances in generalized linear models, which compensates for the decreased robustness with respect to number of clusters [53]. Our results did not change under this compensatory method. Fixed effects included total nodes per network, adult matriline size, average matrilineal coefficient of relatedness, matrilineal rank, and presence of the matriline's matriarch.

Linear mixed-effects regression models (Gaussian distribution) were fit to the three measures of matriline aggression: proportion of aggressive dyadic interactions initiated per matriline using intense aggression, proportion of fighting events in which a matriline participates where the initiator and recipient are kin, and the proportion of fighting events in which the matriline participates and in which intense aggression is involved where the initiator directs intense aggression at kin. Fixed effects included adult matriline size, average coefficient of relatedness within a matriline, matrilineal rank, and presence of the matriline's matriarch. A generalized linear mixed-effects regression model (Poisson distribution) was fit to the counts of wounds/injuries received per matriline. Fixed effects included adult matriline size, the number of communities per matrilineal groom network, number of nodes per matrilineal groom network, matriline rank, and presence of the matriarch. Finally, simple linear regressions were run on the group-level dependent variables and each analysis was limited to a single variable due to small sample size (N = 7 groups). All analyses were performed using Stata (Stata 9; Stata Corporation, College Station, Texas) and the R statistical computing program [49].

## Results

### Matriline-level analyses

#### Community Modularity in Matrilineal Groom Networks

The best fit model included fixed effects for average coefficient of relatedness per matriline and matriline size. However, there were four other models with ΔAIC ≤2, indicating that all five models are equally good at explaining the observed variation in number of communities per matrilineal groom network. Three of these five models included average relatedness as a significant predictor, and four of the five included matriline size as a significant predictor (Table 2).

**Table 2:** Best-fit models for grooming network community structure.

Model	Parameters	AIC	ΔAIC
1	Average matriline relatedness, matriline size	170.7	0.0
2	Matriline size	171.0	0.3
3	Matriline size, nodes	172.0	1.3
4	Average matriline relatedness, matriline size, nodes	172.3	1.6
5	Average matriline relatedness, nodes	172.5	1.8

**Figure 1:**
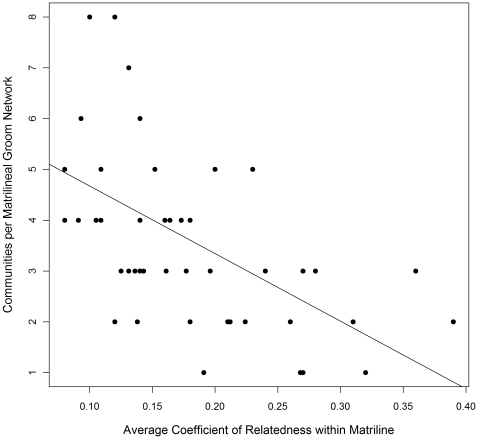
Community structure by average genetic relatedness per matriline. The number of communities per matrilineal groom network is plotted against the matrilineal average coefficient of relatedness for 48 matrilines in seven groups of rhesus macaques. The least-squares regression line is included. Mean matrilineal coefficient of relatedness is 0.18 (range 0.08–0.39), and mean communities per matrilineal groom network is 3.91 (range 1–10).

**Figure 2:**
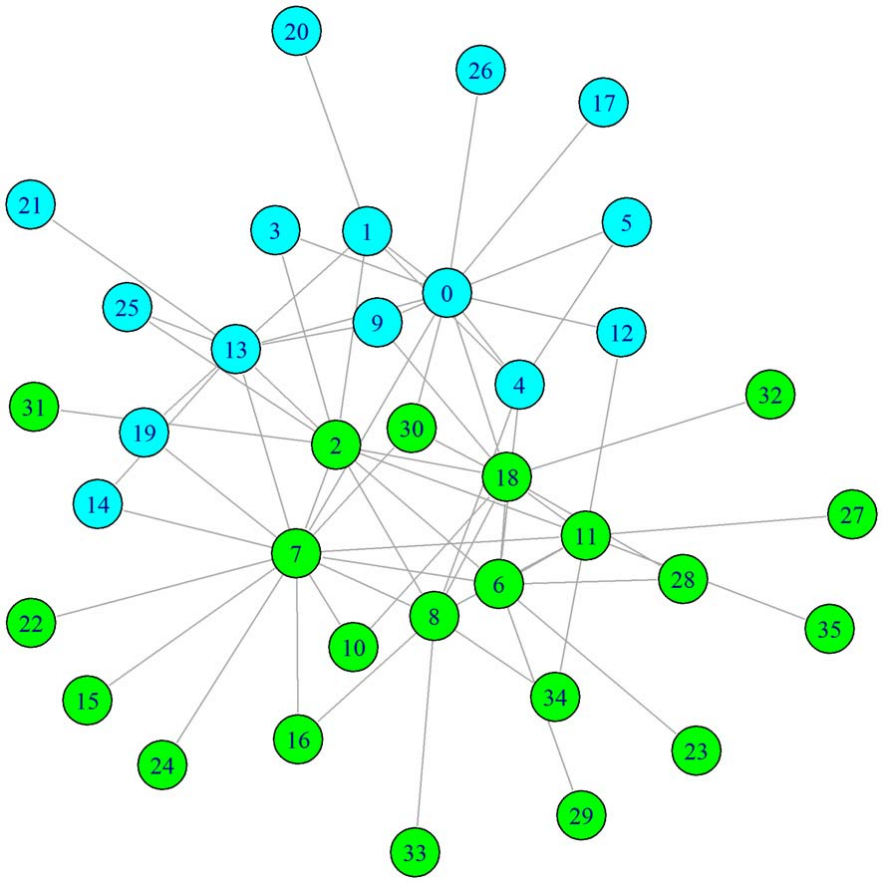
Groom network for matriline D28 in group 14. The groom network for matriline D28 demonstrates a division into two communities (community 1 in cyan; community 2 in green). The D28 matriline pedigree is easily divided into two sub-groups, each of which is descended from one of two sisters (nodes 1 and 2), and the community divisions reflect this genetic fragmentation.

In the best fit model, matrilines having a higher coefficient of relatedness showed significantly fewer communities in their grooming networks (β = −2.05, P<0.0001; Figures 1 and 2). The predicted number of matrilineal groom communities calculated at three different values of average relatedness (minimum, mean, and maximum values for study group matrilines: r = 0.08, r = 0.18, r = 0.39) are 3.9, 3.2, and 2.1 communities, respectively. Additionally, larger matrilines had more communities in their groom networks (β = 0.051, P<0.0001). The predicted number of communities calculated at three different matriline sizes (observed minimum, mean, and maximum adults per matriline: N = 5, N = 10.7, N = 27) are 2.5, 3.3, and 7.6 communities, respectively. The variables matriline size and average coefficient of relatedness per matriline are negatively correlated (r = −0.58).

The remaining four best fit models included the following fixed effects: (2^nd^) matriline size (β = 0.07, P<0.0001), (3^rd^) matriline size (β = 0.05, P<0.0001)and number of nodes per network (β = 0.01, P = 0.002), (4^th^) average matrilineal coefficient of relatedness (β = −1.9, P<0.0001), matriline size (β = 0.04, P<0.0001), and number of nodes per network (β = 0.008, P = 0.01), and (5^th^) average matrilineal coefficient of relatedness (β = −2.6, P<0.0001) and nodes per network (β = 0.01, P<0.0001). These models indicate that, in addition to the average matrilineal coefficient of relatedness and matriline size, the networks with a greater number of nodes had significantly more communities than those with fewer nodes (2^nd^ model: β = 0.010, P = 0.002). The direction and magnitude of the effect of all variables is similar among all five best fit models.

Contrary to expectation, the presence or absence of the matriarch, the most recent maternal common ancestor of all matriline members, did not have a significant influence on the number of communities within the matrilineal groom networks.

#### Proportion of Intense Aggression Initiated per Matriline

**Figure 3:**
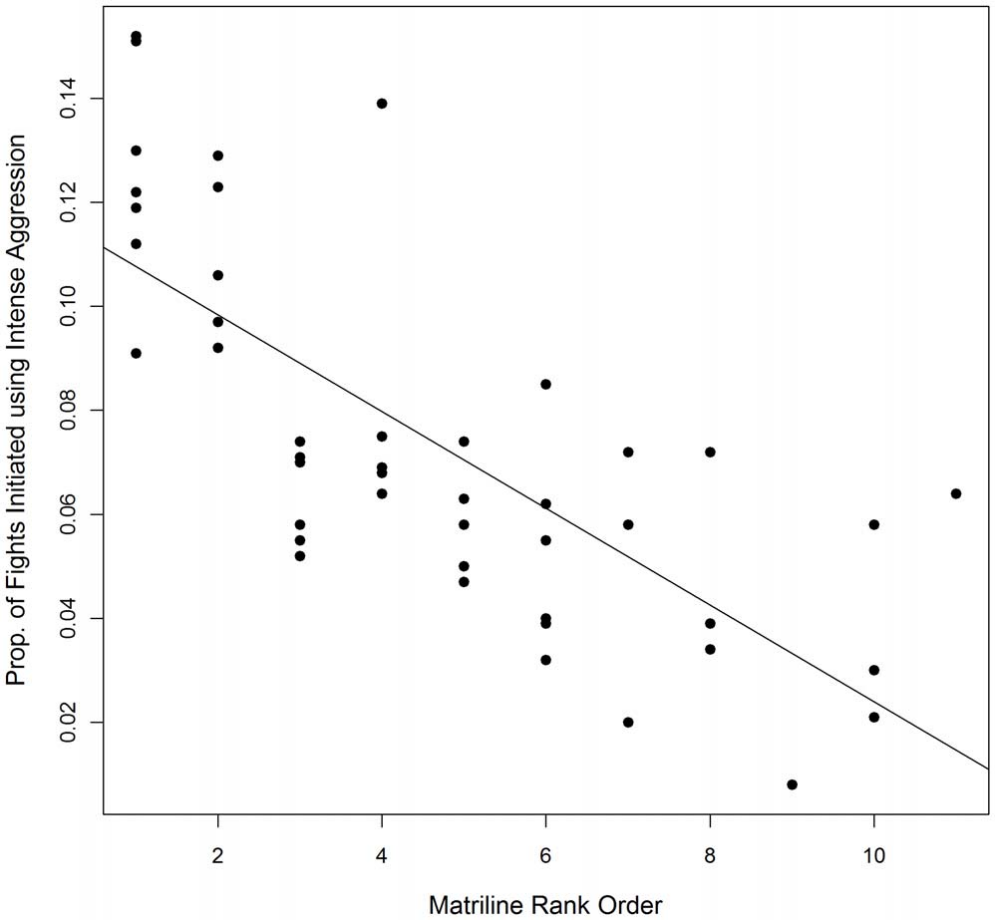
Intense aggression initiated per matriline by matriline rank. The proportion of aggressive dyadic interactions initiated by each matriline using intense aggression plotted against the matriline rank order for 48 matrilines in seven groups of rhesus macaques. The least-squares regression line is included. Mean matrilineal rank is 4.7 (range 1–11) where a rank of 1 represents the highest-ranking matriline.

**Figure 4:**
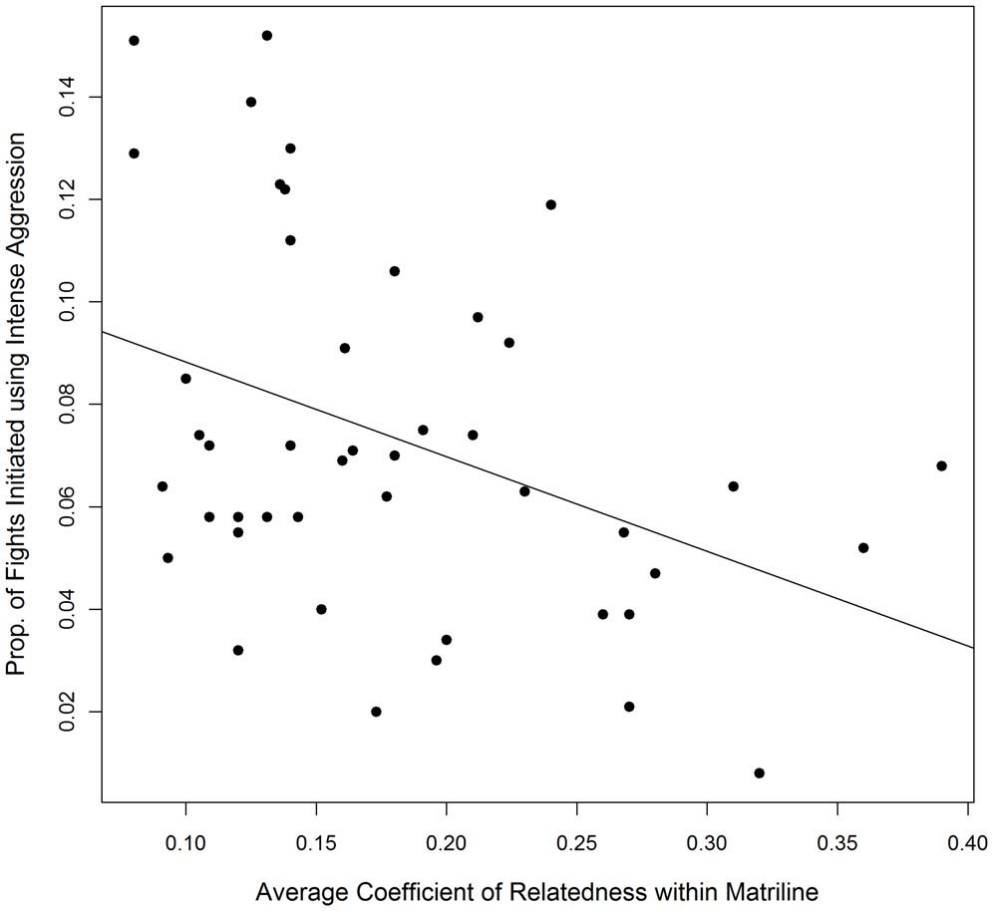
Intense aggression initiated by average genetic relatedness per matriline. The proportion of aggressive dyadic interactions initiated by each matriline using intense aggression plotted against the matrilineal average coefficient of relatedness for 48 matrilines in seven groups of rhesus macaques. The least-squares regression line is included. Mean matrilineal coefficient of relatedness is 0.18 (range 0.08–0.39), and mean proportion of aggressive dyadic interactions initiated using intense aggression per matriline is 0.073 (range 0.008–0.152).

The best fit model included fixed effects for matriline rank and average coefficient of relatedness per matriline (compared to the second and third best fit models, ΔAIC  = 0.50 and ΔAIC  = 2.61, respectively). As expected, higher ranking matrilines initiate a significantly greater proportion of fights using intense aggression than lower ranking matrilines (β = −0.009, P<0.0001; Figure 3). The predicted proportion of fights initiated using intense aggression is 10.5% for alpha matrilines, 7.0% for matrilines ranked fifth in the group, and 2.4% for matrilines ranked tenth in the group, where average coefficient of relatedness is set to the mean value 0.18. Additionally, matrilines having higher average coefficient of relatedness initiate significantly fewer fights using intense aggression than matrilines having lower average coefficient of relatedness (β = −0.15, P = 0.008; Figure 4). The predicted proportion of fights initiated by alpha matrilines using intense aggression, calculated for minimum, mean, and maximum values of average coefficients of relatedness observed in the study groups, are 12.2%, 10.5%, and 6.9%, respectively.

The second best fit model included a fixed effect for matriline rank only, and the direction and magnitude of the effect is similar to the best fit model (β = −0.0096, P<0.0001).

#### Proportion of Fighting-Events between Kin

The best fit model included a fixed effect for average coefficient of relatedness per matriline (compared to the second best fit model, ΔAIC  = 6.50). Matrilines with a lower average coefficient of relatedness participated in a higher proportion of fighting-events in which both combatants were members of that matriline (β = −0.43, P<0.0001). The predicted proportion of fighting-events between kin, calculated for minimum, mean, and maximum values of average coefficients of relatedness observed in the study groups, are 12.2%, 7.9%, and 1%, respectively.

#### Proportion of Fighting-Events with Intense Aggression between Kin

**Figure 5:**
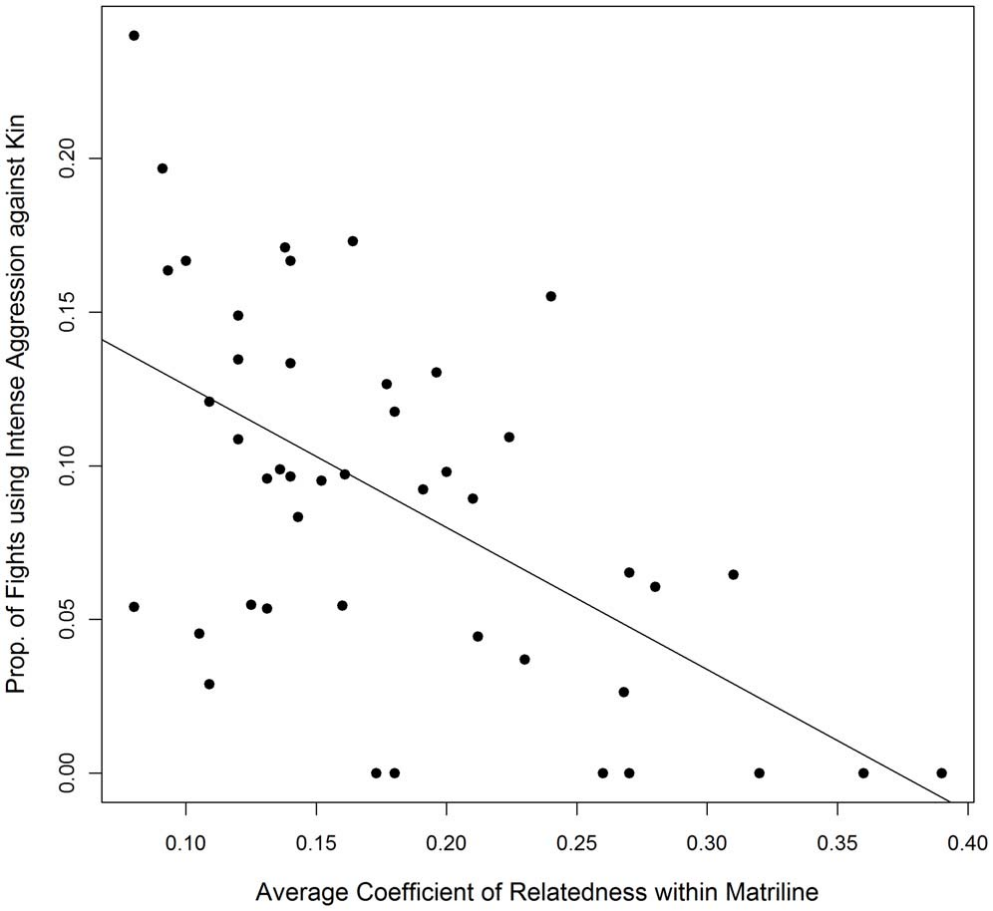
Intense aggression against kin by average genetic relatedness per matriline. The proportion of fighting events involving intense aggression where the initiator directs intense aggression at kin plotted against the matrilineal average coefficient of relatedness for 48 matrilines in seven groups of rhesus macaques. The least-squares regression line is included. Mean matrilineal coefficient of relatedness is 0.18 (range 0.08–0.39), and mean proportion of aggressive dyadic interactions initiated using intense aggression per matriline is 0.089 (range 0–0.24).

The best fit model included a fixed effect for average coefficient of relatedness per matriline (compared to the second best fit model, ΔAIC  = 5.70). As predicted, matrilines with a lower average coefficient of relatedness participated in a higher proportion of fighting-events involving intense aggression in which both combatants were members of that matriline and directed intense aggression at one another (β = −0.48, P<0.0001; Figure 5). The predicted proportion of fighting-events involving intense aggression that are between kin, calculated for minimum, mean, and maximum values of average coefficients of relatedness observed in the study groups, are 13.7%, 8.8%, and <<1%, respectively.

#### Frequency of Wounding Received

The best fit model included fixed effects for the number of communities per matrilineal groom network, number of nodes per matrilineal groom network, adult matriline size, and presence of the matriarch (compared to the second best fit model, ΔAIC  = 5.09). Matrilines having a greater number of communities in their groom networks received wounds and injuries requiring hospitalization more frequently than matrilines having fewer groom communities (β = 0.14, P = 0.001). Matrilineal groom networks having fewer nodes (individuals) received wounds and injuries more frequently than those with more nodes (β = −0.07, P<0.0001). Larger matrilines received more wounds and injuries than smaller matrilines (β = 0.11, P<0.0001). Finally, matrilines whose matriarch was present received fewer wounds and injuries than matrilines whose matriarch was absent (β = −0.57, P = 0.001). The expected number of injuries received per matriline, assuming the matriarch is present and average values for matriline size and number of nodes, is 4.8, 6.4, and 9.7 for networks with 1, 3, and 6 communities, respectively. The expected number of injuries received per matriline, assuming average values for matriline size, number of communities and number of nodes, is 7.3 and 12.9 for matrilines with their matriarch present and absent, respectively.

### Group-level analyses

#### Group-level Average of Communities per Matrilineal Network

We fit a linear regression model to the average value per group (N = 7 groups) of communities per matrilineal groom network using a single predictor: the group-mean average coefficient of relatedness per matriline (which includes all group matrilines weighted by matriline size). This analysis revealed the same relationship between matriline fragmentation and community structure on the group-level as was found for the matriline-level: groups having a higher average value of the average coefficient of relatedness per matriline have a lower average value of communities per matriline (β = −22.4, P = 0.046; R^2^ = 0.69).

#### Group-level Rate of Wounding

We fit a linear regression model to the rate of wounding per group (N = 7 groups) using a single predictor: the group-mean average coefficient of relatedness per matriline. Groups with a higher group-mean average coefficient of relatedness per matriline showed lower rates of wounding (β = −4.11, P = 0.04; R^2^ = 0.52).

#### Group-level Average Coefficient of Relatedness per Matriline

We fit a linear regression model to the group-mean average coefficient of relatedness per matriline using a single predictor: cage age (measured in years). Older cages had significantly lower group-mean average coefficient of relatedness per matriline than younger cages (β = −0.01, P<0.0001; R^2^ = 0.97). The age range observed for the seven study groups is 13.5–32.2 years (mean  = 22.1 years).

## Discussion

The persistence of complex biological systems, such as the persistence of social groups, appears to be dependent upon the presence of factors that promote stability. Kinship is an important organizing principle in many animal societies, frequently structuring the competitive and affiliative relationships among group members, and therefore is a likely source of cohesiveness and stability for many animal societies. We investigated whether kinship structure, created by genetic relationships at the matriline level, is a source of group stability using rhesus macaques as a model species. Overall, our results indicate that lack of close genetic ties at the matriline level is associated with increased sub-grouping within the matriline groom network, more fighting between kin, and more frequent wounding, all of which suggest that genetically fragmented matrilines are less stable than genetically cohesive matrilines.

### Matriline-level instability

Our results show that cohesive matrilineal relationships derived from high average genetic relatedness are a source of stability for rhesus macaque social groups. A low average matrilineal coefficient of relatedness results from the loss of direct genetic links among matriline members, which translates into weakened relationships within the matrilineal groom network, as evidenced by more network communities. This is not surprising, given that grooming and aid in agonistic interactions are patterned by degree of kinship in some macaque species [23], [37]. However, it is surprising that the absence of a matriline's matriarch has no influence on the degree of fragmentation in matrilineal groom networks. Thus a matriarch's presence alone is not sufficient to pull together an otherwise fragmented matriline, nor is the loss of a matriarch sufficient to divide a matriline into subgroups.

The negative relationship between matrilineal genetic relatedness and the initiation of fights using intense aggression indicates that highly fragmented matrilines may possess an unstable social position, as stable dominance hierarchies are maintained with little severe aggression because animals have already sorted out their relative ranks. In addition, the increased fighting among kin when average matrilineal genetic relatedness is low suggests that relative ranks among kin are being contested. Finally, the higher frequency of wounding received by more fragmented matrilines (matriarch absent, low matrilineal genetic relatedness) further supports this relationship between matriline fragmentation and stability of dominance relationships. Although wounding is rarely witnessed during observations, there is a strong implication that fragmented matrilines are targeted with more severe aggression because their social position is not stable, and is therefore contestable.

Our results further support the relationship between social instability and frequency of aggression found in pigtailed macaques. Induction of group instability increased the frequency and intensity of aggression and was associated with smaller, less diverse affiliative networks [6], [8]. In our study groups, a similar pattern emerges at the matriline level: matrilines having lower average coefficients of relatedness exhibit both greater sub-grouping in their affiliation networks as well as increased use of intense aggression, both against others and against kin. This study furthers our understanding of the factors influencing group stability by revealing that instability may originate from genetic fragmentation within kin groups and, in societies where kinship structure is a primary organizing principle, it may be at the level of the matriline that instability has its primary effects.

Our matriline-level results indicate that a high degree of matrilineal genetic relatedness results in cohesive matrilineal relationships that are characterized by less aggression and wounding and integrated grooming ties. Cohesive relationships among kin may serve two functions: (1) to unite kin against non-kin during conflict, which reduces the likelihood that other, lower-ranking matrilines will perceive an opportunity to improve its current social position, and (2) to unite kin such that their unity reduces the likelihood of fighting amongst themselves for higher social rank within the family.

The mechanism by which genetic relationships influence the stability of dominance ranks is through alliances. Inter-matriline ranks as well as individual ranks are maintained by complex networks of alliances among kin as well as among nonkin [12], [54]; it is not simply the largest matriline or individual that is the highest ranking. Increased intense aggression among members of the same matriline is likely an indication that their alliance networks have changed, which may present an opportunity for a female to increase her rank position. In fact, macaque females do appear to take advantage of opportunities to increase their rank, whether naturally occurring or experimentally created. Macaque females will outrank their mother, sisters, a higher-ranking female, or even an entire higher-ranking matriline when given the chance [42], [55], [56], [57]. An opportunity to increase rank is experimentally induced by removing a female's kin allies [42]; in the absence of kin allies, a lower-ranking female and her kin can outrank the formerly higher-ranking female.

### Group-level instability from matriline-level instability

Matriline-level instability appears to translate into group-level instability when multiple matrilines within the group are fragmented. Groups with a lower group-mean matrilineal coefficient of relatedness had higher rates of wounding and more communities per matrilineal groom network, suggesting that there is a cumulative effect of matriline-level instability on group-level instability. Furthermore, high levels of genetic fragmentation within a matriline appear to be related to the age of the group. Among our study groups, those that have been together longer had lower values of group-mean matrilineal coefficient of relatedness. Thus, older groups will have more matrilines which have lost their matriarchs or other adult females that had served as connections between dyads of more distant kin. Genetic fragmentation within a matriline (and within a group) may be an inevitable outcome for an aging group, particularly when group membership cannot be adjusted to new circumstances via fission or dispersal. In human social networks, for example, groups persist for longer when group membership is fluid and individuals have the option of joining or leaving a given group [29]. Therefore, in captive settings where individual animals cannot choose to join or leave a group, preservation of group stability via active group membership adjustment is not an option.

### Group instability and social overthrow

Social groups at their maximum degree of instability are expected to respond to the instability via group fission or social overthrow. In captivity, maximal instability results in a social overthrow, whereby the social hierarchy is disregarded by group members and severe aggression erupts [58]. Social overthrows and fission events have been reported for captive and wild groups of macaques, and a common factor in several of these reports is the sudden absence or incapacitation of the alpha female [57], [59]. Social overthrows in rhesus groups at the CNPRC follow this same pattern [58]. For example, the removal of two alpha females precipitated a social overthrow in two CNPRC groups within 4−10 days of their removal. That the absence of the alpha female can precipitate a social overthrow suggests that: (1) the alpha female's absence fragments the alpha matriline such that instability results and (2) that fragmentation within higher-ranking matrilines is more likely to lead to group instability than fragmentation in lower-ranking matrilines.

Group stability may be robust to the absence of a key adult female or the presence of a single fragmented matriline. However, we suggest that a perturbation to an already fragmented high-ranking matriline or to a group consisting of multiple fragmented matrilines may be the final push toward maximal instability. The social overthrows observed in two CNPRC groups (16 and 12) support this conclusion. In group 16, the alpha matriline average coefficient of relatedness and group-mean matrilineal coefficient of relatedness were the lowest values recorded for all study groups (r = 0.08 and 0.093, respectively), and social overthrow occurred 4 days following the removal of the alpha female (for treatment of conjunctivitis). In group 12, the alpha matriline average coefficient of relatedness was 0.14 (study group mean: r = 0.18), and social overthrow occurred 10 days following the removal of the alpha female (for pregnancy complications). Thus, the sudden absence of a key adult female within an already unstable matriline or group may be a necessary perturbation to precipitate the sufficient degradation of alliance networks or the matrilineal hierarchy such that a social overthrow occurs.

The persistence of stable social groups in primate societies, like the persistence of other complex biological systems, appears to be dependent upon the presence of a number of factors that promote stability. Genetic relationships create a higher-level structure within the group, and this kinship structure has a significant impact on both the affiliative and hierarchical relationships that govern interactions among group members. In general, our results further support that stability in biological systems may stem in large part from the nature of the underlying structures of the system, structures which are characterized by the pattern of relationships among the individual agents of the biological system [1], [3], [8].
